# Absorption and Fluorescence Spectroscopic Properties of 1- and 1,4-Silyl-Substituted Naphthalene Derivatives

**DOI:** 10.3390/molecules17055108

**Published:** 2012-05-03

**Authors:** Hajime Maeda, Tomohiro Maeda, Kazuhiko Mizuno

**Affiliations:** 1 Division of Material Sciences, Graduate School of Natural Science and Technology, Kanazawa University, Kakuma-machi, Kanazawa, Ishikawa 920-1192, Japan; 2 Department of Applied Chemistry, Graduate School of Engineering, Osaka Prefecture University, 1-1 Gakuen-cho, Naka-ku, Sakai, Osaka 599-8531, Japan

**Keywords:** naphthalene, organosilicon compounds, fluorescence, absorption, fluorescence quenching, fluorescence lifetime, fluorescence quantum yield

## Abstract

Silyl-substituted naphthalene derivatives at the 1- and 1,4-positions were synthesized and their UV absorption, fluorescence spectroscopic properties, and fluorescence lifetimes were determined. Analysis of the results shows that the introduction of silyl groups at these positions of the naphthalene chromophore/fluorophore causes shifts of the absorption maxima to longer wavelengths and increases in fluorescence intensities. Bathochromic shifts of the absorption maxima and increases in fluorescence intensities are also promoted by the introduction of methoxy and cyano groups at the naphthalene 4- and 5-positions. In addition, the fluorescence of 9,10-dicyanoanthracene is efficiently quenched by these naphthalene derivatives with Stern-Volmer plot calculated rate constants that depend on the steric bulk of the silyl groups.

## 1. Introduction

Organosilicon compounds are environmentally friendly and generally can be readily synthesized from commercially available halosilanes. The photochemistry of organosilicon compounds [1] has been investigated from the perspective of absorption and emission spectroscopic properties of polysilanes [2], the generation of silylenes and disilenes by photoirradiation to oligosilanes [3,4,5,6,7,8], the formation of intramolecular charge transfer complexes of aromatic disilanes [9,10], and photoinduced electron transfer reactions of organosilicon compounds [11,12,13,14]. In addition, the enhancing effects of silyl groups on the fluorescence intensities of aromatic hydrocarbons, such as anthracenes, naphthacenes, pentacenes, triphenylenes, and pyrenes, has been probed and discussed in detail earlier [15,16,17,18,19,20,21,22,23,24,25,26,27,28,29]. Our previous studies in this area have focused on the effects of silyl, silylethynyl, germyl, and stannyl groups on the absorption and fluorescence spectroscopic properties of pyrene [30,31,32,33,34]. In the photochemistry of silylnaphthalenes, the absorption and fluorescence properties as well as the photoreactions of disilanes [35,36,37,38,39] and oligosilanes [40,41,42] have been subjected to detailed investigation. The results of EPR studies and MO calculations of radical ions generated from silylnaphthalenes have also been described [43,44,45,46,47,48]. In spite of this intense activity, fundamental studies of the absorption and fluorescence properties of monosilyl-group substituted naphthalene derivatives have not been carried out. In the effort described below, the effects of silyl substitution on absorption and fluorescence properties of naphthalene derivatives have been determined, and the steric effects of silyl substituents on quenching of 9,10-dicyanoanthracene fluorescence by naphthalenes has been evaluated.

## 2. Results and Discussion

### 2.1. Synthesis of Silyl-Substituted Naphthalene Derivatives

**Figure 1:**
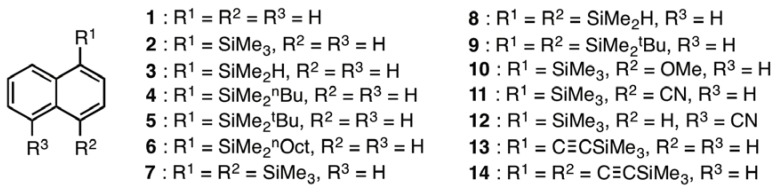
Structures of silyl-substituted naphthalene derivatives.

In order to evaluate the effects of hydrogen atom and alkyl group containing silyl substituents, an acetylene linkage between the naphthalene ring and silyl groups, and the presence of electron donating (OMe) and electron accepting (CN) groups on the absorption and fluorescence properties of naphthalenes, the naphthalene derivatives **2–14** were prepared (Figure 1). The 1-silylnaphthalenes **2–5** were prepared by sequences involving lithiation of 1-bromonaphthalene with *tert*-BuLi followed by addition of the appropriate chlorosilanes. The dimethyl-*n*-octylsilyl group-containing derivative **6** was prepared using a hydrosilylation reaction of 1-octene with the dimethylsilyl substituted naphthalene **3**. The 1,4-disilylnaphthalenes **7–9** were prepared by using an initial dilithiation reaction of 1,4-dibromonaphthalene followed by bis-silylation of the aryl bis-lithium intermediate with chlorosilanes. 1-Bromo-4-methoxynaphthalene was employed as the starting material for the route utilized along with a lithiation-silylation sequence to synthesize the methoxy-substituted derivative **10**. The naphthalene derivatives **11** and **12**, containing 4- and 5-cyano groups respectively, were prepared by employing a cyanation reaction of 1-bromo-4-(trimethylsilyl)naphthalene and a photoinduced electron transfer reaction between **2** and potassium cyanide, respectively. The trimethylsilylethynyl derivatives **13** and **14** were synthesized by using Sonogashira coupling reactions of the corresponding bromonaphthalenes with trimethylsilylacetylene. The detailed synthetic procedures employed to prepare **1–14** are described in the Experimental Section.

### 2.2. Effects of Hydrogen Atom and Alkyl Group Containing Substituents on the Absorption and Fluorescence Properties of Substituted Naphthalenes

**Figure 2:**
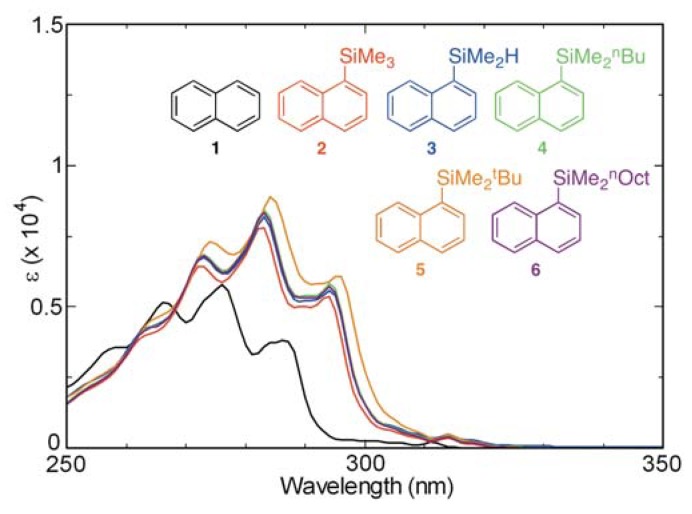
UV absorption spectra of **1–6** in cyclohexane (aerated, *ca.* 10^−4^ M).

**Figure 3:**
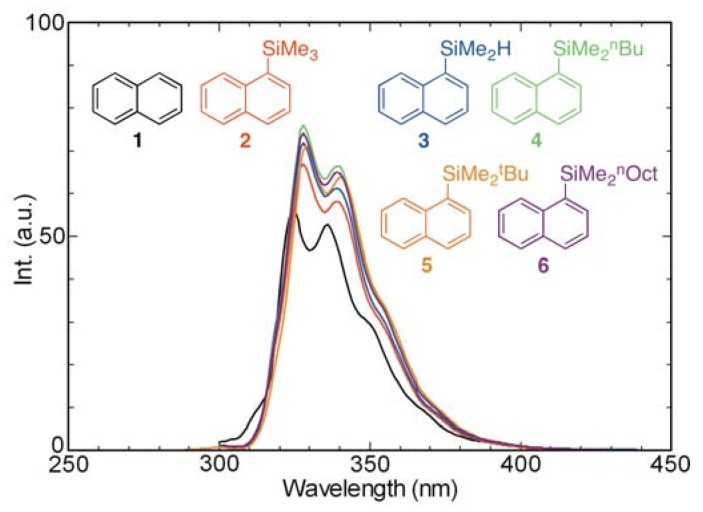
Fluorescence spectra of **1–6** in cyclohexane (aerated, *ca.* 10^−4^ M).

UV-absorption spectra of aerated cyclohexane (*ca.* 10^−4^ M) solutions of the naphthalene derivatives **1–6** are displayed in Figure 2. Evaluation of the spectra shows that the absorption maxima of the silyl-substituted naphthalene derivatives **2–6** shift by 8–9 nm to longer wavelengths and their molecular absorption coefficients (*ε*) increase, compared with those of naphthalene (**1**). Similarly, analysis of the fluorescence spectra of aerated cyclohexane solutions of these compounds (Figure 3) demonstrates that silyl substitution causes emission maxima to shift to longer wavelengths by 4–5 nm and fluorescence intensities to increase, relative to those of naphthalene. Close examination of these spectra shows that substituents on silicon of the silyl groups (SiMe_3_, SiMe_2_H, SiMe_2_^n^Bu, SiMe_2_^t^Bu, and SiMe_2_^n^Oct) have only a small effect on the absorption and fluorescence properties of the naphthalene chromophore/fluorophore. In contrast, silyl-substituents at C-9 of anthracene bring about dramatic changes in absorption and fluorescence properties [20,34]. A possible reason for this phenomenon is steric repulsion, which exists between the C-9 silyl groups and peri-hydrogens at C-1 and C-8 of the anthracene ring. However, this type of effect does normally not occur in naphthalene derivatives, which only possess one peri-hydrogen.

### 2.3. Absorption and Fluorescence Properties of Mono- and Di-silyl Substituted Naphthalene Derivatives

**Figure 4:**
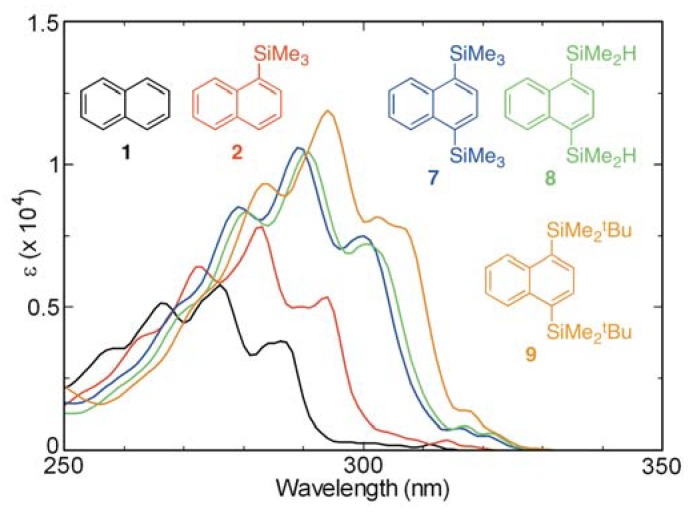
UV absorption spectra of **1**, **2**, and **7–9** in cyclohexane (aerated, *ca.* 10^−4^ M).

**Figure 5:**
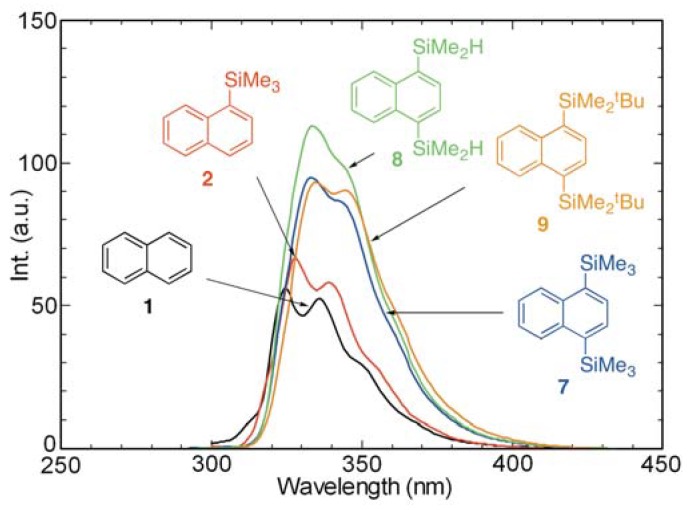
Fluorescence spectra of **1**, **2**, and **7–9** in cyclohexane (aerated, *ca.* 10^−4^ M).

Absorption and fluorescence spectra of cyclohexane solutions of 1,4-bis(trimethylsilyl)naphthalene (**7**), 1,4-bis(dimethylsilyl)naphthalene (**8**), and 1,4-bis(*tert*-butyldimethylsilyl)naphthalene (**9**) were recorded in order to compare their photophysical properties with those of 1-(trimethylsilyl)naphthalene (**2**) and naphthalene (**1**) (Figure 4 and Figure 5). Analysis of the spectra shows that, in general, the absorption maxima of **7–9** occur at longer wavelengths than those of **2** and that the spectrum of the di-*tert*-butyldimethylsilyl derivative **9** displays the most dramatic bathochromic shift. In addition, the fluorescence intensities and emission wavelengths of the disilylnaphthalenes **7–9** are observed to increase relative to those of **1** and **2**.

### 2.4. Effects of Electron-Donating and Withdrawing Groups on Absorption and Fluorescence Properties

**Figure 6:**
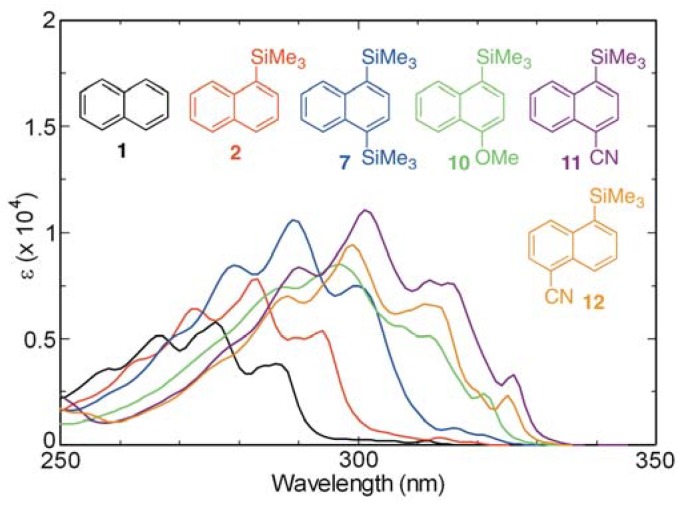
UV absorption spectra of **1**, **2**, **7**, and **10–12** in cyclohexane (aerated, *ca.* 10^−4^ M).

**Figure 7:**
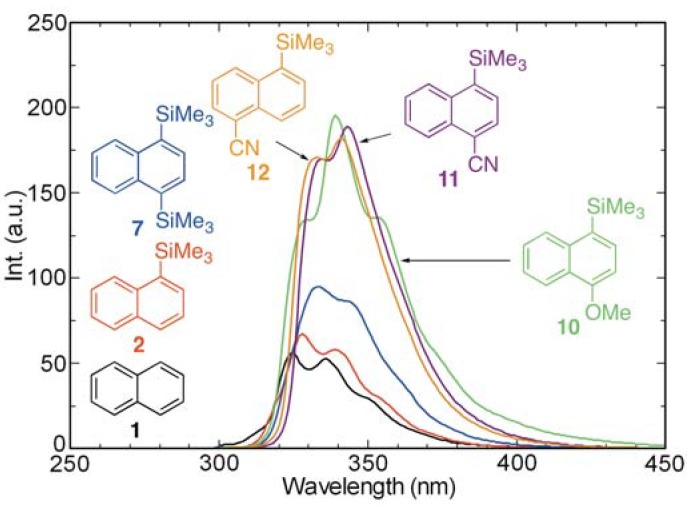
Fluorescence spectra of **1**, **2**, **7**, and **10–12** in cyclohexane (aerated, *ca.* 10^−4^ M).

Since the presence of electron-donating and withdrawing groups can dramatically influence the photophysical properties of organic chromophores/fluorophores, we investigated the influence of such inductive effects by various substituent groups on the absorption and fluorescence properties of 1-(trimethylsilyl)naphthalenes. For this purpose, absorption and fluorescence spectra recorded using cyclohexane solutions of cyanonaphthalenes **11** and **12** and methoxynaphthalene **10** were compared with those of naphthalene and its 1-trimethylsilyl (**2**) and 1,4-bis-trimethylsilyl (**7**) derivatives (Figure 6 and Figure 7). As can be deduced from the recorded spectra, the presence of the 4-methoxy (**10**), 4-cyano (**11**), and 5-cyano (**12**) groups results in long wavelength shifts of the absorption maxima and slight increases in the molar absorption coefficients (*ε*), which is in stark contrast to those of the parent arene. Moreover, the fluorescence intensities of **10–12** are larger than those of **1**, **2**, and **7**.

### 2.5. Effects of Silylethynyl Group(s) on Absorption and Fluorescence Properties

**Figure 8:**
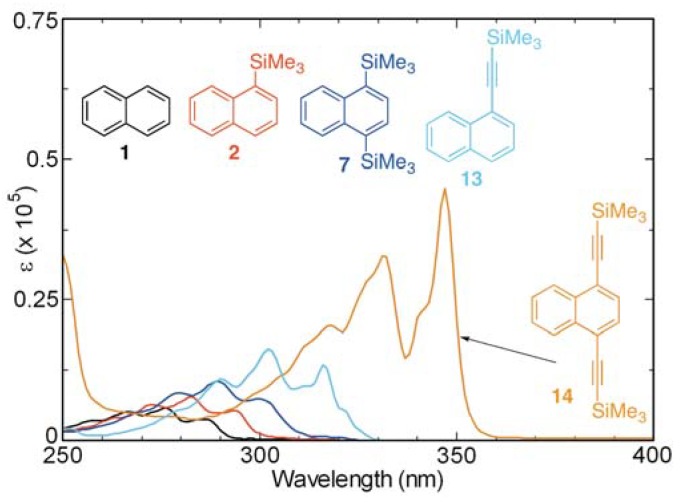
UV absorption spectra of **1**, **2**, **7**, **13**, and **14** in cyclohexane (aerated, 10^−4^–10^−5^ M).

**Figure 9:**
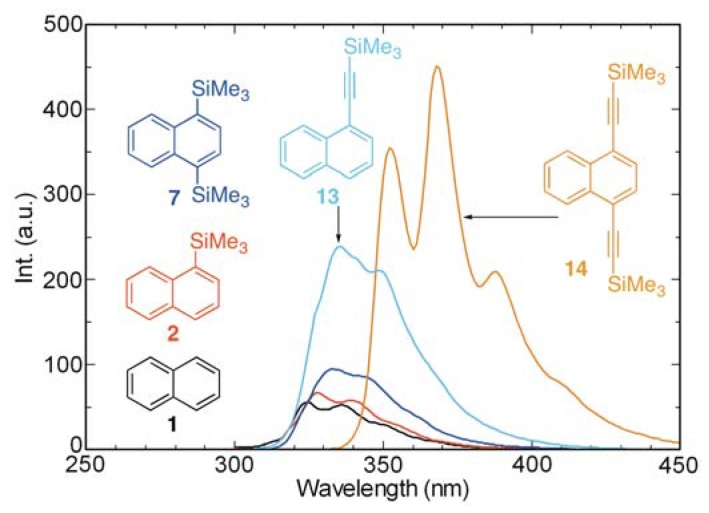
Fluorescence spectra of **1**, **2**, **7**, **13**, and **14** in cyclohexane (aerated, 10^−4^–10^−5^ M).

The results of recent studies have shown that the silylethynyl group(s) brings about bathochromic shifts of the maxima in the absorption spectra of aromatic substances and that these groups lead to increases in fluorescence intensities [24,27,28,29,31]. Consequently, we probed the influence of the silylethynyl group(s) on the absorption and fluorescence properties of naphthalenes **13** and **14**, and found that the absorption maxima of the naphthalene chromophore/fluorophore shift to longer wavelengths and *ε* increases as a consequence of the presence of silylethynyl group(s) (Figure 8). Especially noteworthy is the observation that, in the 1,4-bis-(trimethylsilylethynyl) derivative **14**, the absorption maximum is strongly bathochromically shifted to 347 nm and *ε* increases to 4.4 × 10^4^ mol^−1^dm^3^cm^−1^ in comparison with those of other substances studied. Moreover, the fluorescence intensities of the silylethynyl-substituted naphthalenes **13** and **14** are also larger than the other naphthalene derivatives (Figure 9).

### 2.6. Fluorescence Quenching of 9,10-Dicyanoanthracene by Silylnaphthalenes

**Figure 10:**
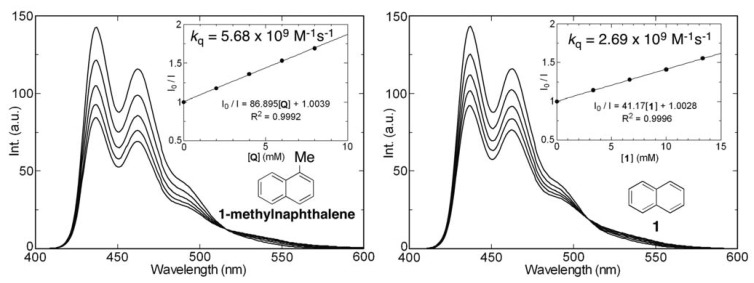

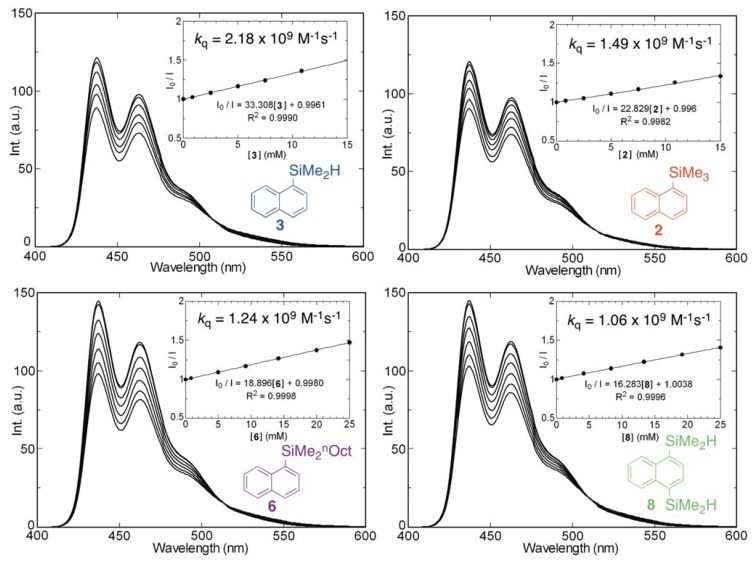
Quenching of the fluorescence of 9,10-dicyanoanthracene (aerated benzene, *λ*_ex_ = 426 nm) by naphthalene derivatives. Insets are Stern-Volmer plots determined by using fluorescence intensities at *λ*_ex_ = 437 nm.

Experiments were carried out to evaluate how the electronic and steric effects of silyl groups govern the ability of naphthalene derivatives to serve as quenchers of the singlet excited state of 9,10-dicyanoanthracene. For this purpose, fluorescence emission intensities of benzene solutions of 9,10-dicyanoanthracene in the presence of various concentrations of 1-methylnaphthalene and the naphthalene derivatives **1–3**, **6** and **8** were determined (Figure 10). The rate constants of 9,10-dicyanoanthracene fluorescence quenching, calculated by using Stern-Volmer analysis and falling in range of 5.68 × 10^9^ to 1.06 × 10^9^ M^−1^s^−1^, were observed to decrease in the following order: 1-methylnaphthalene > **1** > **3** > **2** > **6** > **8**. 

Additionally, analysis of the fluorescence profiles shows that weak emission from singlet exciplexes, formed between 9,10-dicyanoanthracene and the naphthalene derivatives, occurs at longer wavelengths. The results indicate that the rate constant for fluorescence quenching is dependent on the steric bulk and not on the electronic effects of the silyl substituents present in the naphthalene quenchers.

### 2.7. HOMO-LUMO Energy Calculations, Fluorescence Lifetimes, and Fluorescence Quantum Yields

The results described above demonstrate that the introduction of silyl group(s) promotes a shift in the maxima and an increase in molar absorption coefficients (*ε*) in the absorption spectra of naphthalenes (Table 1). Bathochromic shifts and incremental increases of *ε* were also observed when electron-withdrawing, electron-donating, and silylethynyl groups are attached to the naphthalene ring. In order to understand these effects, the HOMO and LUMO energies of **1**–**14** were calculated using the PM3 method. Inspection of the results shows that the HOMO and LUMO energy gaps in these arenes decrease in the following order: **1** > **2**–**6** > **7**–**9** > **10**–**12** > **13**–**14**, a finding that is in good agreement with the experimental absorption spectroscopic results. Specifically, introduction of silyl groups causes an increase in both the HOMO and LUMO energies of naphthalenes and a net overall decrease in energy gaps between these orbitals. In this regard, silyl substituents act as electron-donating groups as a consequence of *σ*(C-Si)-π*(naphthalene) molecular orbital interactions. Because introduction of silylethynyl group(s) significantly lowers the energy of the naphthalene LUMO, both σ*(C-Si)-*π*(naphthalene) and *σ*(C-Si)-π*(naphthalene) orbital interactions are taking place in these substances. 

Since both the fluorescence lifetime and the quantum yield are important physical properties of fluorophores, we determined these parameters for a number of the naphthalene compounds synthesized. Fluorescence lifetimes (*τ*_s_) of the naphthalenes were determined in both aerated and carefully degassed cyclohexane solutions. The accumulated data show that the fluorescence lifetimes of these substances decrease in the following order: **1** > **2–6** > **7–9** > **10–12** > **13–14**. In addition, the large differences observed between the fluorescence lifetimes of aerated and degassed solutions indicate that molecular oxygen has a significant fluorescence quenching effect. 

Finally, the fluorescence quantum yields (*Φ*_f_) of selected members of the series of naphthalenes explored in this investigation were determined in thoroughly degassed solutions to exclude the influence of dioxygen. The data produced, which included quantum yield values of *Φ*_f_ 0.30 (**2**), 0.33 (**7**), 0.65 (**10**), 0.66 (**11**), and 0.85 (**14**), demonstrate that these substances fluoresce significantly more efficiently than naphthalene (*Φ*_f_ = 0.23 [49]).

## 3. Experimental 

### 3.1. General

Purifications of solvents were carried out in the following manner: acetonitrile was distilled from P_2_O_5_ and then from CaH_2_. Benzene was distilled from CaH_2_ and then from Na. Isopropanol was distilled from CaH_2_. Piperidine was distilled from KOH. THF was distilled from CaH_2_, and then from Na and Ph_2_C=O. Other chemicals were used as purchased.

Melting points were determined on a Yanagimoto Yanaco MP-500 Micro Melting Point apparatus, and are uncorrected. ^1^H- and ^13^C-NMR spectra were recorded using a Varian MERCURY-300 (300 MHz and 75 MHz, respectively) spectrometer with Me_4_Si as an internal standard. Mass spectra (CI) were recorded utilizing a JEOL JMS-DX303 spectrometer. High-performance liquid chromatographic (HPLC) separations were performed using a recycling preparative HPLC, equipped with a Jasco PU-2086 Plus pump, RI-2031 Plus differential refractometer, Megapak GEL 201F columns (GPC) with CHCl_3_ as the eluent. HPLC separations (packed silica gel) were performed by a recycling preparative HPLC equipped with Jasco PU-987 pump, UV-970 uv-vis detector, CHEMCOSORB I-5Si column (Chemco Scientific Co., Ltd.) with hexane and EtOAc as eluents. Column chromatography was conducted by using MERCK silica gel 60 (0.063–0.200 mm). Thin layer chromatography (TLC) was performed using MERCK silica gel 60 F_254_.

UV-vis and fluorescence spectra were recorded using Jasco UV-160 and FP-770 spectrophotometers, respectively, employing a 1 cm pathlength cell at 298 K. Solutions of the naphthalenes in spectral grade cyclohexane (*ca.* 10^−4^ M (**1–12**) or 10^−5^ M (**13**, **14**)) were prepared under aerated conditions. Excitation wavelengths were the longest wavelength absorption maxima unless otherwise noted and absorbances at the excitation wavelengths were 0.5. Fluorescence lifetimes were measured by using a HORIBA NAES-550 nano-second fluorometer equipped with a SSU-111A photomultiplier, SCU-121A optical chamber, SGM-121A monochromator, and LPS-111 lamp power supply. All decay curves were fitted by utilizing a single exponential decay with chi square values less than 2. Fluorescence quantum yields (*Φ*_f_) were determined using carefully degassed (freeze-pump-thaw method) cyclohexane solutions. Standards for fluorescence quantum yields are listed in the footnotes of Table 1. Fluorescence quenching experiments were performed by adding benzene solutions of quenchers (1.00 M (1-methylnaphthalene and **1**) or 0.25 M (**2**, **3**, **6**, and **8**)) to 10^−5^ M benzene solutions of 9,10-dicyanoanthracene (*λ*_ex_ = 426 nm). Stern-Volmer plots were obtained by using fluorescence intensities at *λ*_ex_ = 437 nm and the reported fluorescence lifetime of 9,10-dicyanoanthracene (*τ*_s_ = 15.3 ns [50]).

### 3.2. Preparation of Silyl-Substituted Naphthalene Derivatives

*1-(Trimethylsilyl)naphthalene* (**2**). A *tert*-butyllithium (1.49 M in *n*-pentane, 30.0 mL, 44.7 mmol) solution was slowly added to argon-purged THF solution (100 mL) of 1-bromonaphthalene (2.80 mL, 20.0 mmol) at −78 °C. The resulting solution was stirred for 30 min at −78 °C before adding Me_3_SiCl (7.6 mL, 60.1 mmol). Subsequently, the solution was warmed to room temperature and stirred overnight before slowly adding saturated aqueous NaHCO_3_. The Et_2_O layer obtained by extraction was dried over Na_2_SO_4_, filtered, and concentrated *in vacuo* to give a residue which was subjected to column chromatography on silica gel (eluent: hexane) followed by HPLC (GPC). These procedures yielded 1-(trimethylsilyl)naphthalene (**2**, 96% yield) as a colorless liquid, ^1^H-NMR (CDCl_3_) *δ* 0.47 (s, 9 H), 7.42–7.53 (m, 3 H), 7.69 (dd, *J* = 1.28, 6.78 Hz, 1 H), 7.83–7.88 (m, 2 H), 8.08–8.11 (m, 1 H).

*1-(Dimethylsilyl)naphthalene* (**3**). This substance was prepared by using a similar procedure as employed in the preparation of **2**, starting with 1-bromonaphthalene (2.80 mL, 20.0 mmol), *tert*-butyllithium (1.49 M in *n*-pentane, 28.6 mL, 42.6 mmol), Me_2_HSiCl (6.7 mL, 60.3 mmol) and THF (150 mL). Purification of the product by using column chromatography on silica gel (eluent: hexane, *R*_f_ = 0.5–0.6) followed by HPLC (GPC) produced 1-(dimethylsilyl)naphthalene (**3**, 88% yield) as a colorless liquid; ^1^H-NMR (CDCl_3_) *δ* 0.49 (d, *J* = 3.85 Hz, 6 H), 4.82–4.90 (m, 1 H), 7.45–7.55 (m, 3 H), 7.72 (dd, *J* = 1.37, 6.68 Hz, 1 H), 7.84–7.89 (m, 2 H), 8.10–8.14 (m, 1 H).

*1-(n-Butyldimethylsilyl)naphthalene* (**4**). This substance was prepared by using a similar procedure as employed in the preparation of **2**, starting with 1-bromonaphthalene (4.2 mL, 30.0 mmol), *n*-butyllithium (1.5 M in *n*-hexane, 37.5 mL, 56.3 mmol), Me_2_HSiCl (10.0 mL, 90.0 mmol) and THF (150 mL). GC-Mass analysis of the crude product mixture indicated that it contained 1-(dimethylsilyl)naphthalene (**3**, 81%) and 1-(*n*-butyldimethylsilyl)naphthalene (**4**, 16%). Separation by using column chromatography on silica gel (eluent: hexane) followed by HPLC (GPC) yielded 1-(*n*-butyldimethylsilyl)naphthalene (**4**) as a colorless liquid; ^1^H-NMR (CDCl_3_) *δ* 0.45 (s, 6 H), 8.82–0.87 (m, 3 H), 0.95–1.00 (m, 2 H), 1.29–1.35 (m, 4 H), 7.41–7.52 (m, 3 H), 7.67 (dd, *J* = 1.28, 6.78 Hz, 1 H), 7.83–7.87 (m, 2 H), 8.07–8.10 (m, 1 H).

*1-(tert-Butyldimethylsilyl)naphthalene* (**5**). This substance was prepared by using a similar procedure as employed in the preparation of **2**, starting with 1-bromonaphthalene (1.40 mL, 10.0 mmol), *tert*-butyllithium (1.51 M in *n*-pentane, 15.0 mL, 22.7 mmol), ^t^BuMe_2_SiCl (3.8 g, 25.2 mmol) and THF (100 mL). Purification by using column chromatography on silica gel (eluent: hexane, *R*_f_ = 0.7) followed by HPLC(GPC) gave 1-(*tert*-butyldimethylsilyl)naphthalene (**5**, 43% yield) as a colorless solid; mp 75–77 °C; ^1^H-NMR (CDCl_3_) *δ* 0.50 (s, 6 H), 0.94 (s, 9 H), 7.42–7.47 (m, 3 H), 7.70 (dd, *J* = 1.28, 6.96 Hz, 1 H), 7.82–7.86 (m, 2 H), 8.10–8.14 (m, 1 H).

*1-(Dimethyl-n-octylsilyl)naphthalene* (**6**). An *i*-PrOH (0.15 mL) solution containing H_2_PtCl_6_•6H_2_O (5.0 mg, 0.009 mmol) and 1-octene (1.57 mL, 10.0 mmol) was heated to 70 °C before slowly adding 10 mL of a benzene solution of 1-(dimethylsilyl)naphthalene (**3**, 1.590 g, 8.5 mmol). After stirring the solution at reflux for 24 h, benzene and water were added. Extraction with benzene gave an organic layer that was dried over Na_2_SO_4_ and concentrated *in vacuo*. Subjection of the residue to column chromatography on silica gel (eluent: hexane, *R*_f_ = 0.6–0.7) followed by HPLC(GPC) gave 1-(dimethyl-*n*-octylsilyl)naphthalene (**6**, 55% yield) as a colorless liquid; ^1^H-NMR (CDCl_3_) *δ* 0.44 (s, 6 H), 0.85 (t, *J* = 6.78 Hz, 3 H), 0.94–1.06 (m, 2 H), 1.21–1.30 (m, 12 H), 7.41–7.53 (m, 3 H), 7.67 (dd, *J* = 1.28, 6.78 Hz, 1 H), 7.83–7.87 (m, 2 H), 8.07–8.10 (m, 1 H).

*1,4-Dibromonaphthalene.* To a stirred CCl_4_ mixture (150 mL) naphthalene (**1**, 12.817 g, 100 mmol) and iron powder (59 mg, 1.1 mmol), a CCl_4_ solution (20 mL) of Br_2_ (10.3 mL, 201 mmol) was slowly added under stirring. The resulting mixture was stirred vigorously for 25 h at room temperature. Concentrated HCl was slowly added until the pH of the mixture reached 7. Extraction with Et_2_O gave an organic layer that was dried over Na_2_SO_4_ and concentrated *in vacuo* to give a solid. Recrystallization from EtOH (twice) yielded 1,4-dibromonaphthalene (56% yield) as a colorless solid; ^1^H-NMR (CDCl_3_) *δ* 7.64 (s, 2 H), 7.65 (dd, *J* = 3.48, 6.41 Hz, 2 H), 8.26 (dd, *J* = 3.20, 6.50 Hz, 2 H). 

*1,4-Bis(trimethylsilyl)naphthalene* (**7**). A *tert*-butyllithium (1.49 M in *n*-pentane, 23.5 mL, 35.0 mmol) solution was slowly added to as argon-purged THF solution (150 mL) containing 1,4-dibromo-naphthalene (2.000 g, 7.0 mmol) at −78 °C. After stirring for 30 min, Me_3_SiCl (5.3 mL, 42.0 mmol) was added at −78 °C and the resulting solution was warmed to room temperature and stirred overnight. Slow addition of saturated aqueous NaHCO_3_ followed by extraction with Et_2_O gave an organic layer that was dried over Na_2_SO_4_ and concentrated *in vacuo*. The residue was subjected to column chromatography on silica gel (eluent: hexane, *R*_f_ = 0.65) followed by recrystallization from MeOH and yielded 1,4-bis(trimethylsilyl)naphthalene (**7**, 80% yield) as a colorless solid; mp 88–89 °C; ^1^H-NMR (CDCl_3_) *δ* 0.46 (s, 18 H), 7.50 (dd, *J* = 3.48, 6.41 Hz, 2 H), 7.66 (s, 2 H), 8.13 (dd, *J* = 3.30, 6.41 Hz, 2 H).

*1,4-Bis(dimethylsilyl)naphthalene* (**8**). This substance was prepared analogously to **7**, starting with 1,4-dibromonaphthalene (503 mg, 1.8 mmol), *tert*-butyllithium (1.49 M in *n*-pentane, 5.0 mL, 7.5 mmol), Me_2_HSiCl (1.2 mL, 10.8 mmol) and THF (100 mL). Purification of the product by using column chromatography on silica gel (eluent: hexane, *R*_f_ = 0.6) followed by HPLC(GPC) gave 1,4-bis(dimethylsilyl)naphthalene (**8**, 70% yield) as a colorless liquid; ^1^H-NMR (CDCl_3_) *δ* 0.49 (d, *J* = 3.85 Hz, 12 H), 4.85 (m, 2 H), 7.53 (dd, *J* = 3.30, 6.41 Hz, 2 H), 7.70 (s, 2 H), 8.14 (dd, *J* = 3.30, 6.41 Hz, 2 H).

*1,4-Bis(tert-butyldimethylsilyl)naphthalene* (**9**). This substance was prepared analogously to 7, starting with 1,4-dibromonaphthalene (1.400 g, 4.9 mmol), *tert*-butyllithium (1.51 M in *n*-pentane, 8.2 mL, 12.4 mmol), ^t^BuMe_2_SiCl (2.183 g, 14.5 mmol) and THF (100 mL). Purification of the product by using column chromatography on silica gel (eluent: hexane, *R*_f_ = 0.8) followed by HPLC(GPC) and recrystallization from MeOH gave 1,4-bis(*tert*-butyldimethylsilyl)naphthalene (**9**, 12% yield) as colorless needle-like crystals; mp 100–101 °C; ^1^H-NMR (CDCl_3_) *δ* 0.50 (s, 12 H), 0.95 (s, 18 H), 7.43 (dd, *J* = 3.39, 6.50 Hz, 2 H), 7.65 (s, 2 H), 8.12 (dd, *J* = 3.39, 6.50 Hz, 2 H).

*1-Methoxy-4-(trimethylsilyl)naphthalene* (**10**). To a CCl_4_ solution (50 mL) of 1-methoxynaphthalene (1.597 g, 10.1 mmol), a CCl_4_ solution (20 mL) of Br_2_ (0.47 mL, 9.2 mmol) was slowly added under stirring. After stirring the solution vigorously for 25 h at room temperature, concentrated HCl was added slowly until the pH of the mixture reached 7. Extraction with Et_2_O gave an organic layer that was dried over Na_2_SO_4_ and concentrated *in vacuo* to give crude 1-bromo-4-methoxynaphthalene (2.373 g, ca 10.0 mmol) as a yellow liquid. To an argon-purged THF solution (100 mL) of crude 1-bromo-4-methoxynaphthalene (2.373 g, ca 10.0 mmol), *tert*-butyllithium (1.40 M in *n*-pentane, 14.3 mL, 20.0 mmol) was slowly added at −78 °C. After stirring the solution for 30 min, Me_3_SiCl (3.8 mL, 30.1 mmol) was added at −78 °C. The resulting solution was warmed to room temperature, stirred overnight and diluted by addition of saturated aqueous NaHCO_3_. Extraction with Et_2_O gave an organic layer that was dried over Na_2_SO_4_ and concentrated *in vacuo*. The residue was subjected to column chromatography on silica gel (eluent: hexane/EtOAc = 9/1, *R*_f_ = 0.8–0.9) followed by HPLC (GPC) to give 1-methoxy-4-(trimethylsilyl)naphthalene (**10**, 65% yield based on 1-methoxynaphthalene) as a colorless liquid; ^1^H-NMR (CDCl_3_) *δ* 0.44 (s, 9 H), 4.00 (s, 3 H), 6.80 (d, *J* = 7.69 Hz, 1 H), 7.44–7.54 (m, 2 H), 7.60 (d, *J* = 7.69 Hz, 1 H), 8.02–8.06 (m, 1 H), 8.31–8.34 (m, 1 H).

*1-Cyano-4-(trimethylsilyl)naphthalene* (**11**). To a an argon-purged THF solution (150 mL) of 1,4-dibromonaphthalene (1.400 g, 4.9 mmol), *tert*-butyllithium (1.51 M in *n*-pentane, 3.9 mL, 5.9 mmol) was slowly added at −78 °C. After stirring for 30 min, Me_3_SiCl (0.75 mL, 5.9 mmol) was added at −78 °C. The resulting solution was warmed to room temperature and stirred overnight. Saturated NaHCO_3_ aqueous solution was slowly added. Extraction with Et_2_O gave an organic layer that was dried over Na_2_SO_4_ and concentrated *in vacuo*. Subjection of the residue to column chromatography on silica gel (eluent: hexane, *R*_f_ = 0.8) gave crude 1-bromo-4-(trimethylsilyl)naphthalene (991 mg, *ca.* 3.5 mmol) as a colorless liquid. A solution containing *N*-methyl-2-pyrrolidone (NMP, 50 mL), crude 1-bromo-4-(trimethylsilyl)naphthalene (991 mg, ca 3.5 mmol), and CuCN (310 mg, 3.5 mmol) was stirred at reflux (*ca.* 200 °C) for 15 h, cooled to room temperature, and diluted by the addition of aqueous NH_3_, benzene, and Et_2_O. The organic layer was separated, washed with dilute aqueous NH_3_ solution (twice), H_2_O (twice), and brine (twice), subsequently dried over Na_2_SO_4_, and concentrated in vacuo. Subjection of the residue to column chromatography on silica gel (eluent: benzene, *R*_f_ = 0.75) followed by HPLC(GPC) yielded 1-cyano-4-(trimethylsilyl)naphthalene (**11**, 30% yield) as a colorless liquid; ^1^H-NMR (CDCl_3_) *δ* 0.49 (s, 9 H), 7.61–7.72 (m, 2 H), 7.72 (d, *J* = 7.14 Hz, 1 H), 7.86 (d, *J* = 7.14 Hz, 1 H), 8.16–8.19 (m, 1 H), 8.28–8.31 (m, 1 H).

*1-Cyano-5-(trimethylsilyl)naphthalene* (**12**) [51,52]. Dioxygen was bubbled for 20 min through a solution of KCN (195 mg, 3.0 mmol), 1-(trimethylsilyl)naphthalene (2, 60 mg, 0.3 mmol), and 9-cyanophenanthrene (8 mg, 0.04 mmol) in H_2_O (0.5 mL) and CH_3_CN (3 mL) in a cyrindrical Pyrex glass vessel (*φ* = 8 mm, *l* = 20 cm). This solution was irradiated for 10 h at room temperature with a 300 W high-pressure mercury lamp (Eikosha, PIH-300), under continuous aeration with dioxygen. The temperature of the solution was kept constant arround room temperature during irradiation with a water cooling system. After the reaction was completed (as monitored by TLC), benzene, Et_2_O, and brine were added. The separated organic phase was dried over Na_2_SO_4_ and concentrated *in vacuo*, giving a residue that was subjected to column chromatography on silica gel (eluent: toluene, *R*_f_ = 0.5–0.6) followed by HPLC (GPC) and HPLC (packed silica gel) to yield 1-cyano-5-(trimethylsilyl)naphthalene (**12**, 35% yield) and 1-cyano-4-(trimethylsilyl)naphthalene (**11**, 36% yield). Data for **12**: ^1^H-NMR (CDCl_3_) *δ* 0.48 (s, 9 H), 7.55 (dd, *J* = 7.23, 8.51 Hz, 1 H), 7.65 (dd, *J* = 6.78, 8.42 Hz, 1 H), 7.82 (dd, *J* = 1.28, 6.78 Hz, 1 H), 7.92 (dd, *J* = 1.10, 7.14 Hz, 1 H), 8.27–8.36 (m, 2 H).

*1-(Trimethylsilylethynyl)naphthalene* (**13**). A N_2_-purged solution containing 1-bromonaphthalene (2.8 mL, 20.0 mmol), trimethylsilylacetylene (3.3 mL, 23.9 mmol), Pd(PPh_3_)_2_Cl_2_ (565 mg, 0.8 mmol), CuI (82 mg, 0.4 mmol), and piperidine (50 mL) was stirred for 2 h at room temperature and then at 80 °C overnight. After cooling to room temperature, piperidine was removed by distillation under reduced pressure. Concentrated HCl was added to the residue and the solution was extracted with CHCl_3_. The separated organic phase was dried over Na_2_SO_4_ and concentrated *in vacuo*, giving a residue that was subjected to column chromatography on silica gel [eluent: gradient from hexane to hexane/benzene = 4/1, *R*_f_ = 0.6 (hexane)] followed by HPLC (GPC) to yield 1-(trimethylsilylethynyl)naphthalene (**13**, 43% yield) as a colorless liquid; ^1^H-NMR (CDCl_3_) *δ* 0.34 (s, 9 H), 7.40 (dd, *J* = 7.14, 8.24 Hz, 1 H), 7.48–7.60 (m, 2 H), 7.70 (dd, *J* = 1.10, 7.14 Hz, 1 H), 7.80–7.85 (m, 2 H), 8.33 (dd, *J* = 0.73, 8.24 Hz, 1 H).

*1,4-Bis(trimethylsilylethynyl)naphthalene* (**14**). A N_2_-purged solution containing 1,4-dibromo-naphthalene (2.860 g, 10.0 mmol), trimethylsilylacetylene (3.4 mL, 24.6 mmol), Pd(PPh_3_)_2_Cl_2_ (560 mg, 0.8 mmol), CuI (76 mg, 0.4 mmol), and piperidine (50 mL) was stirred for 2 h at room temperature and then at 80 °C overnight. After cooling to room temperature, piperidine was removed by distillation under reduced pressure. Concentrated HCl was added to the residue and the solution was extracted with CHCl_3_. The separated organic phase was dried over Na_2_SO_4_ and concentrated *in vacuo*, giving a residue that was subjected to column chromatography on silica gel [eluent: gradient from hexane to hexane/benzene = 4/1, *R*_f_ = 0.4 (hexane)] followed by HPLC (GPC) and recrystallization from MeOH to yield 1,4-bis(trimethylsilylethynyl)naphthalene (**14**, 95% yield) as a colorless solid; mp 73.5–75 °C; ^1^H-NMR (CDCl_3_) *δ* 0.33 (s, 18 H), 7.61 (dd, *J* = 3.30, 6.41 Hz, 2 H), 7.62 (s, 2 H), 8.33 (dd, *J* = 3.11, 6.41 Hz, 2 H); ^13^C-NMR (CDCl_3_) *δ* -14.72, 86.42, 87.79, 106.50, 111.53, 112.24, 114.96, 118.02; MS (CI), *m*/*z* (%) = 73 (1), 305 (4), 321 (M^+^+1, 100). Anal. Calcd for C_20_H_24_Si_2_: C, 74.93; H, 7.55. Found: C, 74.90; H, 7.40.

## 4. Conclusions

In the study described above, the absorption and fluorescence properties of 1-silyl-, 1,4-disilyl-, 1-silylethynyl- and 1,4-disilylethynyl-naphthalenes were evaluated. The findings show that the absorption maxima of the 1-silyl- and 1,4-disilylnaphthalenes occur at longer wavelengths with larger *ε* values than those of naphthalene (**1**). Bathochromic effects and incremental increases in *ε* were also observed for electron-donating, electron-withdrawing and silylethynyl group substituted naphthalenes. The results of PM3 calculations of HOMO-LUMO energy gaps are in accord with the experimental observations. Furthermore, fluorescence quantum efficiencies were found to increase and fluorescence lifetimes decrease when the silyl substituents are present on the naphthalene ring system. Also, the respective fluorescence quantum yield and lifetime of 1,4-bis(trimethylsilylethynyl)naphthalene (**14**) were found to be 0.85 and 2 ns. Finally, the fluorescence of 9,10-dicyanoanthracene is efficiently quenched by silylnaphthalenes, with quenching rate constants that depend on the steric bulk and not the electronic properties of these substituents.

## Figures and Tables

**Table 1:** Photophysical properties of **1**–**14**.

	R^1^	R^2^	R^3^	Absorption ^a^			Eigenvalues ^b^ (eV)				fluorescence					Quenching ^e^
				*λ*_abs_ (nm)	log *ε*		HOMO	LUMO	energy gap		*λ*_em_^a^ (nm)	*τ*_s_^c^ (aerated) (ns)	*τ*_s_^d^ (degassed) (ns)	*Φ*_f_		*k*_q_ (M^−1^s^−1^)
**1**	H	H	H	286	3.58		–8.765	–0.467	8.298		324	16	96^f^	0.23^f^		2.69 × 10^9^
2	SiMe_3_	H	H	294	3.73		–8.543	–0.345	8.198		328	15	64	0.30^g^		1.49 × 10^9^
3	SiMe_2_H	H	H	294	3.75		–8.568	–0.362	8.206		328	15	61	–^i^		2.18 × 10^9^
4	SiMe_2_^n^Bu	H	H	294	3.74		–8.560	–0.361	8.199		329	15	63	–^i^		–^i^
5	SiMe_2_^t^Bu	H	H	295	3.78		–8.557	–0.361	8.196		329	15	56	–^i^		–^i^
6	SiMe_2_^n^Oct	H	H	294	3.76		–8.562	–0.363	8.199		329	16	62	–^i^		1.24 × 10^9^
7	SiMe_3_	SiMe_3_	H	300	3.87		–8.341	–0.250	8.091		333	14	23	0.33^g^		–^i^
8	SiMe_2_H	SiMe_2_H	H	300	3.86		–8.385	–0.280	8.105		333	13	30	–^i^		1.06 × 10^9^
9	SiMe_2_^t^Bu	SiMe_2_^t^Bu	H	302	3.91		–8.369	–0.286	8.083		335	12	–^i^	–^i^		–^i^
10	SiMe_3_	OMe	H	312	3.71		–8.260	–0.260	8.000		327	6	10	0.65^g^		–^i^
11	SiMe_3_	CN	H	315	3.88		–8.876	–0.956	7.920		333	8	11	0.66^g^		–^i^
12	SiMe_3_	H	CN	311	3.82		–8.894	–0.959	7.935		333	8	–^i^	–^i^		–^i^
13	C≡CSiMe_3_	H	H	316	4.13		–8.487	–0.584	7.903		336	9	–^i^	–^i^		–^i^
14	C≡CSiMe_3_	C≡CSiMe_3_	H	347	4.65		–8.236	–0.714	7.522		352	2	2	0.85^h^		–^i^

^a^ [**1-12**] = ~1 × 10^−4^ M in cyclohexane. [**13-14**] = ~1 × 10^−5^ M in cyclohexane; ^b^ Calculated by PM3; ^c^ Aerated, 10^−5^–10^−4^ M in cyclohexane, **1–8**, **10**, and **12** were excited at peak top of absorption band appeared at the longest wavelength. Compounds **9** and **14** were excited at 265 nm. Compound **11** was excited at 312 nm. Compound **13** was excited at 302 nm. Filter glass used was UV-32; ^d^ Degassed by freeze-pump-thaw method, 10^−5^–10^−4^ M in cyclohexane. Compounds **1–4**, **6-12** were excited at 265 nm. Compound **5** was excited at 254 nm. Filter glass used was UV-32; ^e^ Fluorescence quenching of 9,10-dicyanoanthracene (aerated, ~1 × 10^−5^ M in benzene, 3.0 mL, excited at 426 nm, Stern-Volmer plot: 437 nm, *τ*_s_ = 15.3 ns by silylnaphthalenes; ^f^ Value in reference 49; ^g^ Degassed by freeze-pump-thaw method, 10^−5^–10^−4^ M in cyclohexane, excited at 265 nm (**1**, **2**: Abs = 0.5 at 265 nm, **7**, **10**, and **11**: Abs = 0.05 at 265 nm). Reference compound was naphthalene (**1**); ^h^ Degassed by freeze-pump-thaw method, ~5 × 10^−6^ M in cyclohexane, excited at 323 nm (Abs = 0.1 at 323 nm). Reference compound was anthracence (*Φ*_f_ = 0.36, Data from reference 49); ^i^ Not measured.
